# Depressive disorder in childhood: The importance of an early diagnosis for a functional recovery. Specific symptoms and treatment in an 8-years old patient with depression

**DOI:** 10.1192/j.eurpsy.2021.891

**Published:** 2021-08-13

**Authors:** S.S. Sánchez Rus, M.O. Solis, L. Soldado Rodriguez, M. Suárez-Gómez

**Affiliations:** 1 Psychiatrist, Mental Health Unit, Ugc Jaén, University Hospital, Complejo Hospitalario Jaén, Jaén, Spain; 2 Jaén, Complejo Hospitalario Jaén, Jaén, Spain; 3 Mental Health Unit, Complejo Hospitalario de Jaen, Jaen, Spain; 4 Psychiatry, ULSBA - Hospital José Joaquim Fernandes, Beja, Portugal

**Keywords:** Depression, childhood, functional recovery, early diagnosis

## Abstract

**Introduction:**

Depressive disorders (Dd) in childhood have a prevalence about 1-2%. Sometimes depression may be underdiagnosed with the risk of complications: comorbidity, chronicity or development of psychiatric diseases in adulthood. Although children often do not show a clear sad mood, they usually presents irritability as a cardinal symptom. Other common symptoms in children´s depression are lack of attention, difficult of concentration and impulsivity. These symptoms actually could define as well an Attention Deficit and Hyperactivity Disorder (ADHD), highly prevalent in school-aged children (5-7%).

**Objectives:**

-To deep into diagnosis and evolution of depressive disorder in primary school-aged children (7-12 years-old). -To contrast clinical evidence about specific aged-symptoms observed in the boy and follow-up until remission.

**Methods:**

-Case study. Graphic description of diagnosis path and treatment in a 8-years-old boy suffers from depression. -Clinical case attended in Mental Health Unit, ambulatory consultation (outpatient). -Diagnosis tools: Clinical examination, family interview, evaluation tests and school psychopedagogical assessment.

**Results:**

-Treatment methods: psychotherapy, psychopharmacology and theater. -Specific depressive symptoms depends on childhood stages (*chart by ages). -Pharmacological treatment used: psychostimulants, benzodiazepines and antidepressants. -Efficacy of monotherapy with Fluoxetine 20mg/day 6-months. -Importance of individual psychotherapy and group activities 12-months. -Episode resolution and functional recovery 15-months.

**Conclusions:**

Variability of symptoms in children´s depression can be confused with other psychiatric disorders like decreased school performance (ADHD), that may make diagnosis difficult. Sometimes, both disorders coexist, especially when the mood disorder is secondary to academic problems caused by ADHD. Early diagnosis and continued follow-up in specialized units is necessary to avoid progression and complications of Dd.

